# Evaluation of the Effect of Polyurea Coating Application on the Capacity and Deformability of Reinforced Concrete Beams

**DOI:** 10.3390/ma18245674

**Published:** 2025-12-17

**Authors:** Artur Matusiak, Tomasz Waśniewski, Jacek Szafran

**Affiliations:** 1 Department of Structural Mechanics, Faculty of Civil Engineering, Architecture and Environmental Engineering, Lodz University of Technology, Aleje Politechniki 6, 93-590 Lodz, Poland; artur.matusiak@p.lodz.pl (A.M.); jacek.szafran@p.lodz.pl (J.S.); 2 Department of Concrete Structures, Faculty of Civil Engineering, Architecture and Environmental Engineering, Lodz University of Technology, Aleje Politechniki 6, 93-590 Lodz, Poland

**Keywords:** polyurea, reinforced, concrete, bending, parametric, analysis

## Abstract

Polyurea coatings as a possible structural reinforcement system is a research project involving the investigation of the potential of using polyurea coatings to improve the performance characteristics of structures (steel, concrete, timber and others used in construction). This study, which is part of the aforementioned project, focuses on evaluating the effect of polyurea coating application on the strength and deformability parameters of reinforced concrete elements. For the purposes of this publication, nonlinear cross-sectional analyses were carried out using a layered model, and the model was then calibrated and verified against experimental results. The study was supplemented by a numerical analysis of a reinforced concrete beam coated with polyurea, and the adopted numerical model was calibrated and verified using experimental results. For the calibration and verification of the computational models, experimental tests of reinforced concrete beams with a low degree of reinforcement (0.7%), were used. The results of the numerical analyses are consistent with those of the experimental studies, and an increasing effect of the polyurea coating is demonstrated with thicker layers of its application. Numerical analysis shows that the reinforcement ratio had a strong influence on the effectiveness of the polyurea coating.

## 1. Introduction

Polyurea coating products were developed and marketed in the United States in the 1980s. In Europe, these products began to appear in the second half of the 1990s and have seen significant development since the beginning of the 21st century. Initially, polyurea coatings were used to protect polyurethane foam, mainly in the thermal insulation of roofs. Technologically, they are composite materials applied using suitable machines (spraying units) to spray two-component materials, hot and under pressure, as coating insulation. Polyurea, from a material point of view, is an elastomer formed by a chemical reaction (polyaddition) between an aromatic or aliphatic isocyanate and a mixture of amines. Aromatic polyureas are products in which the isocyanate components are prepolymers made from methylenediphenyl diisocyanate (MDI), while aliphatic products are formed from hexamethylene diisocyanate (HDI) or isophorone diisocyanate (IPDI), which form the hard part of the chain structure. Aromatic polyureas are widely used in the construction industry for waterproofing roofs and foundations, anti-corrosion and chemical-resistant protection and waterproofing and sealing coatings [1,2,3,4,5,6,7,8,9,10].

Research on polyurea, which started at the turn of the 20th century, mainly involved analyses of the basic properties of this material. Publications [11,12,13,14,15] focused on the analysis of the basic properties of the coating, especially its elasticity in tensile tests. Studies [16,17,18,19] described the effect of varying environmental conditions (e.g., high temperature) on the change in coating properties. Another series of articles describes possible applications of polyurea and composite materials in ballistic systems and devices. Papers [20,21,22,23,24] analysed the effects of polyurea coating on ballistic components (such as helmets, aluminium and steel plates). Articles [25,26,27,28,29] focused on how composite (polymer) materials protect non-standard structural components from the effects of explosives. In contrast to the numerous studies and papers on the properties of polyurea coatings, only a few publications can be found in the literature that focus on the assessment of the performance of structural components. Studies [30,31,32,33,34,35] describe the results of research and evaluation of the effect of polyurea coating application on the performance of selected components used in construction (such as wooden joints, water pipes, steel plates and concrete coils). More information covering current research directions of coatings is available in the doctoral dissertation [1]. However, the available studies do not provide a comprehensive explanation of how the application of polyurea coatings affects the strength and deformability parameters of reinforced concrete elements.

The present study, which includes nonlinear analysis of the flexural section (using a layered model) and numerical analyses of reinforced concrete beams coated with polyurea coatings, aims to extend this knowledge. Experimental studies of reinforced concrete beams with a low degree of reinforcement (0.7%), which formed one of the components of the doctoral thesis [1], were used to calibrate and verify the computational models.

A parametric analysis was performed to evaluate the interaction of the polyurea coating thickness with other parameters such as the degree of reinforcement, the yield strength of steel and the compressive strength of concrete.

### Research Significance

Concrete and reinforced concrete structures are constantly exposed to corrosive factors (chemical, biological and atmospheric corrosion). There are many techniques for repairing and strengthening reinforced concrete elements. First and foremost, there are traditional techniques involving reinforcement by adding additional external reinforcement, overconcreting, etc. There are also methods using composite materials laminated to the concrete surface or bonded into the concrete cover.

Polyurea coating systems are most frequently and readily used for repairing concrete and reinforced concrete structures. Preliminary analyses and experimental tests have shown that polyurea application affects the performance of such elements under load. For fully informed use of these systems, it is necessary to determine their impact on the strength parameters of the elements they coat. A good solution is to present simple computational models for determining the effect of polyurea application on the performance characteristics of elements under load. The experimental tests and development of the computational model used reinforced concrete elements, for which the largest number of polyurea sprays are currently implemented.

The presented research and analysis aim to evaluate the behaviour of beams coated with polyurea. The use of polymer as a reinforcing layer may have additional benefits. Firstly, the polyurea coating layer is resistant to highly aggressive environments found, for example, in the chemical industry, food processing and sewage treatment plants. In these cases, the use of traditional methods may not produce the expected results due to overly aggressive external factors.

Secondly, due to its method of application, the polyurea coating bonds very well with the substrate, which is important in the case of repairs to severely degraded surfaces.

Polyurea coatings are a class of elastomeric copolymers which have a unique set of mechanical properties and offer a wide range of applications in construction. The use of polyurea coating in recent decades has been widely practised in modern engineering constructions. It is caused by unusual properties of the coating, such as high elasticity of the material, scratch-resistance or very high adhesion to many surfaces. The polyurea can be applied to prolong the operation of many existing engineering objects through improving their resistance against external factors (chemical and biological corrosion or atmospheric factors).

An analysis of the impact of polyurea coatings will allow for a measurable assessment of the applicability of this type of solution and identify its limitations.

## 2. Experimental Investigations [1,36,37]

The studies carried out in [1,36,37] were used as the basis for the computational analysis. These studies focused on the assessment of load-carrying capacity and behaviour under load of beams with rectangular cross-section. The studies included 6 beams: 3 elements without coating and 3 elements with coating. The variable parameter was the tensile reinforcement ratio of the beams.

### 2.1. Geometry and Reinforcement of the Specimens

Experimental tests were carried out on six reinforced concrete beams with a length of 3.6 m. The beams were tested in a four-point loading scheme. The support spacing was 3.2 m. All beams had the same rectangular cross-section with dimensions of 15 cm × 30 cm and were made of the same concrete.

Two upper bars with a diameter of 10 mm and two bottom bars with a diameter of 14 mm were used, giving a reinforcement ratio of *ρ_s_* = 0.7%.

The stirrups were made of 6 mm diameter bars and spaced every 150 mm, with a density of 100 mm at the supports. Ribbed steel reinforcement bars with a nominal characteristic yield strength of *f_yk_* = 500 MPa were used.

**Figure 1:**
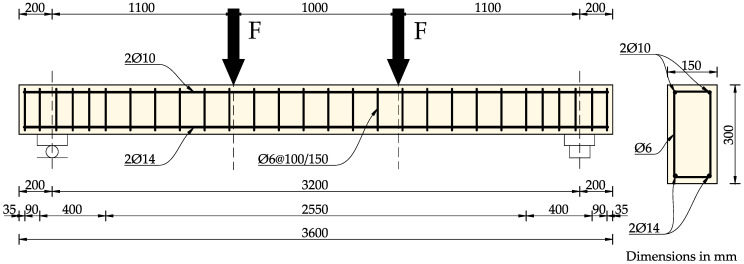
Dimensions and reinforcement of the specimens. Drawn based on [1,36,37].

The dimensions and layout of the reinforced concrete beams are shown in Figure 1.

The complete series of the specimens (6 reinforced concrete beams) was divided into two sets: Set I—reference beams without coating, marked as B.2.1, B.2.2 and B.2.3; Set II—beams coated with polyurea and marked as P.2.1, P.2.2 and P.2.3.

**Figure 2:**
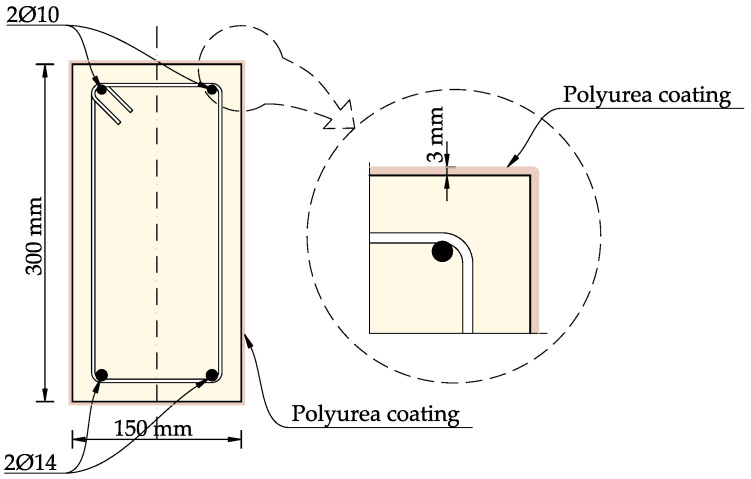
Polyurea-coated elements—arrangement of the coating according to [1,36,37].

**Figure 3:**
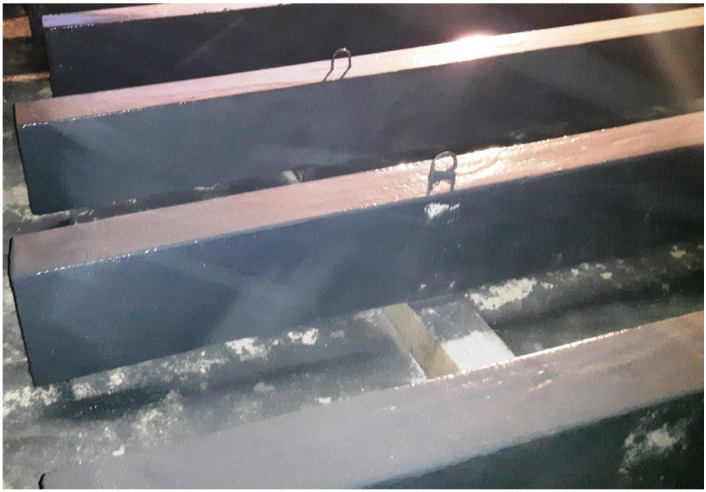
Polyurea-coated elements [1,36,37].

The three specimens of the RC beams were polyurea-coated on all of their outer surfaces. The details of the polyurea coating are shown in Figure 2, and the coated beams are shown in Figure 3.

The polyurea application process consisted of three main phases: concrete preparation, prime coat application and polyurea application. The first phase involved checking the elements and looking for the presence of substances inhibiting the coating adherence. Next, the RC beams were polished and cleaned. The second phase involved applying the specific epoxy-resin-based primer and dry quartz sand to achieve the best coating adhesion. The third phase involved spraying a coating. The polyurea coating was applied in two layers: the first layer on the concrete surface, the second layer directly on the first layer, perpendicular to the direction in which the first layer was applied. This approach made it possible to produce a fully continuous coating with an average thickness of 2.5–3.0 mm, according to the material manufacturer’s guidelines. During all three phases, substrate and ambient temperatures as well as humidity were controlled to provide optimal conditions for this process. More details and a full description of each phase of the polyurea application process are shown in [1,36,37].

### 2.2. Materials

All beams were made of concrete from a single batch. The concrete mix recipe consisted of 610 kg/m^3^ of fine sand with a grain size of 0–2 mm, 470 kg/m^3^ of 2–8 mm gravel, 690 kg of 8–16 mm gravel, 400 kg/m^3^ of Portland cement CEM I 42.5 and 100 L/m^3^ of water. In addition, plasticiser and superplasticiser admixtures were added.

As part of the experimental research [1,36,37], the strength parameters of the concrete used were verified at the time of testing. These values were obtained on the basis of cubic (cube) concrete samples with a side length of 150 mm. The concrete compressive and tensile strength tests were carried out in accordance with European standards [38,39]. The strength parameters of the concrete are summarised in Table 1.

The basic strength parameters of the reinforcing bars used were verified as part of the experimental tests. The strength characteristics of the reinforcing steel used were determined in accordance with the requirements of [40]. A summary of the strength parameters of the top and bottom reinforcing rebars is presented in Table 2.

The experimental studies used aromatic polyurea, whose isocyanate component consists of prepolymers based on methylene diphenyl diisocyanate (MDI). The basic mechanical parameters were obtained on the basis of paddle-shaped samples made from the supplied polyurea components in uniaxial tensile strength. The tests were determined in accordance with EN ISO 527:2012 [41]. The two load speeds were assumed: 50 mm/min and 100 mm/min. The obtained parameters of polyurea are summarised in Table 3.

### 2.3. Test Setup

The specimens were loaded using a steel crossbeam, which was placed symmetrically relative to the beam axis. This allowed for a setup in the form of a four-point loading system. The spacing between the forces is 100 cm, creating a pure bending zone in the middle of the specimen. The tested elements were placed on a horizontal steel frame structure with two supports, with an axial spacing of 320 cm. One support was designed as a fixed (non-sliding) type, while the other was configured as a sliding type to prevent unintended axial forces.

The test and measurement system was equipped with a set of 12 sensors to measure the displacement of reinforced concrete beams. The set included 10 LVDT and 2 dial gauges (marked as S-1 and S-2). A computer workstation equipped with the necessary software and a multi-channel measurement system was used to control the measurements and record the readings from the sensors.

**Figure 4:**
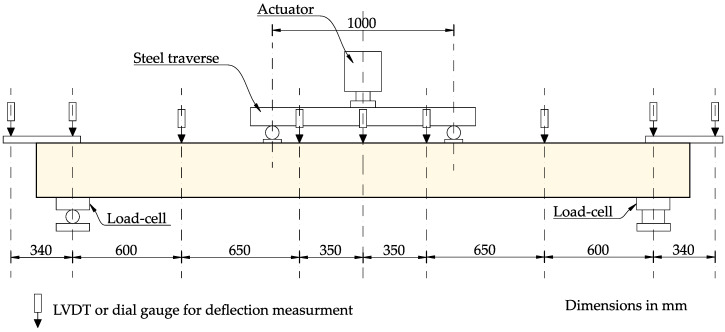
The test stand for testing RC beams. Drawn based on [1,36,37].

An overview of the test setup and measurement layout is presented in Figure 4.

### 2.4. Loading Procedure and Measurements

Loads were applied to the specimens in steps. In the case of beams without a coating load was applied until it failed. No unloading was carried out. In case of specimens covered with polyurea coating, a loading–unloading process was carried out to assess the residual strains and deflections in those types of elements.

Specimens were loaded with stepwise increments of vertical force with values of 10 kN (in the initial loading phase) and 5 kN (in the final loading phase). The given force value was determined on the basis of the readings of the hydraulic piston power supply device and verified using the values read from two load cells placed under the supports.

During the loading of the beam elements, the vertical displacements, the force exerted by the hydraulic piston and the reaction value on one of the supports were registered. The beam deflection values were verified using an LVDT mounted on an independent steel frame (Figure 4). The readings of the inductive sensors and force gauges were recorded automatically at a specified load level with a recording frequency of 0.2 s.

More information and details on the experimental research can be found in [1,36,37].

## 3. Test Results

All specimens failed as expected. In both the reference beams and those coated with polyurea, failure occurred due to crushing of the concrete in the compression zone, preceded by yielding of the tensile reinforcement.

**Figure 5:**
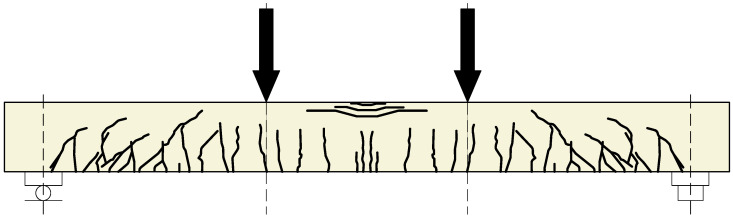
The cracking pattern of the RC beam without polyurea coating [1,36,37].

**Figure 6:**
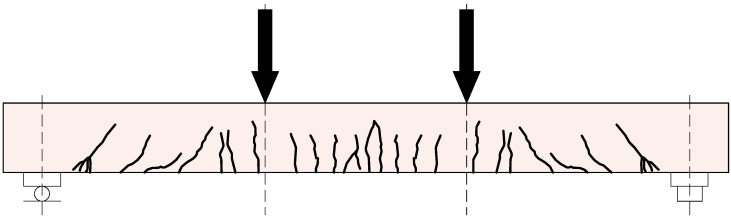
The cracking pattern of the polyurea-coated RC beam.

In all specimens, a typical crack pattern for elements in flexure developed, with a tendency for cracks in the middle of the span. As the load increased, the deflection of the beams and the number of cracks increased. In accordance with the adopted static loading scheme for the elements, numerous vertical cracks were observed in the middle of the beam span (Figure 5 and Figure 6)—in the pure bending zone. Outside this area, diagonal cracks were also identified, which were mainly concentrated in the support zones of the elements.

It should be noted that the polyurea coating delayed the cracking of the beams to some extent, but did not significantly change the morphology of the cracks in the bending and shear zones. Polyurea coating effectively bridged the surface cracking to such an extent that it was visible (to the naked eye) only for large crack widths.

Table 4 summarises the results of the experimental tests [1,36,37].

**Figure 7:**
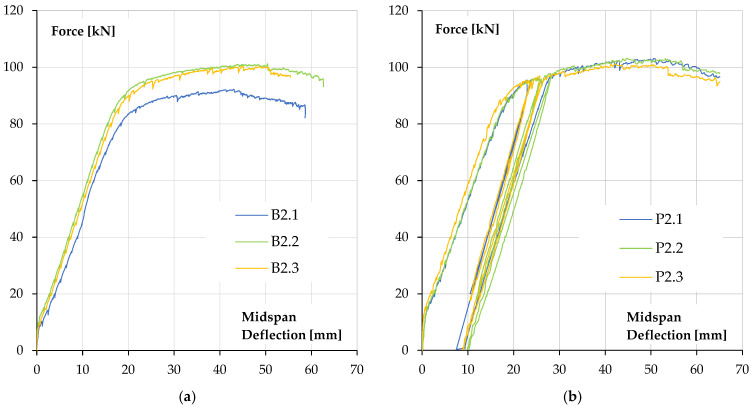
Force–deflection at midspan relationships of tested specimens: (**a**) reference beams, (**b**) beams coated with polyurea coating [1,36,37].

The relationship between force (the force exerted by the actuator) and beam deflection, measured at its mid-span, is shown in Figure 7.

The obtained results of ultimate forces and bending moments show a slight increase in the values of it for reinforced concrete beams covered with a polyurea coating in relation to uncoated specimens. An increase in the ultimate force is about 9.2% (5.5 kN·m). The increase in the load bearing capacity of reinforced concrete elements should be considered insignificant and unsatisfactory in terms of the costs of applying the coating, which must be incurred for the purpose of applying polyurea. However, this solution may be an alternative to repairing existing reinforced concrete elements for which traditional reinforcement is not possible. In the case of beams coated with polyurea, it was possible to carry out a loading–unloading process, in which the start of unloading was set at a load level of 90% of the ultimate loading force for the reference beam. It is worth noting that the secondary load bearing capacity (bending load bearing capacity after the loading–unloading process) of reinforced concrete beams coated with polyurea was achieved without excessive increase in the deflection of these elements—loss of bending stiffness (Figure 7).

## 4. Numerical Analysis

### 4.1. Model and Materials

A numerical model was created using OpenSees v.3.1 software (open source, developed at the University of California, Berkeley, CA, USA) [42], which was developed to simulate the nonlinear response of structural elements.

The beam was idealised using one-dimensional “nonlinearBeamColumn” finite elements with five integration points, taking into account material and geometric nonlinearities.

The stiffness at the integration points was determined from the cross-sectional N-M-κ constitutive relationship determined from the cross-sectional layer model.

The following assumptions are made in the presented calculation approach:

- Only normal stresses are considered;
- The principle of sectional plane conservation is valid over the entire load range;
- The loading is monotonic, unidirectional and irreversible;
- The material relations σ-ε are determined for an uniaxial stress state;
- The strain of the reinforcement and the surrounding concrete are the same in both the compression and tension zones;
- The concrete in compression is described by a nonlinear relation according to [43];
- Concrete in tension is described by a nonlinear relationship, taking into account tension-stiffening [44,45];
- The polyurea layer is described by an idealised relationship based on data obtained in a uniaxial tension test [1];
- The reinforcement steel is described by a material model according to [46];
- The strain of the concrete and the polyurea layer is the same in both the compression and tension zones due to the strong adhesion of the polyurea coating to the concrete surface. This is confirmed by experimental observations [1,36,37].

**Figure 8:**
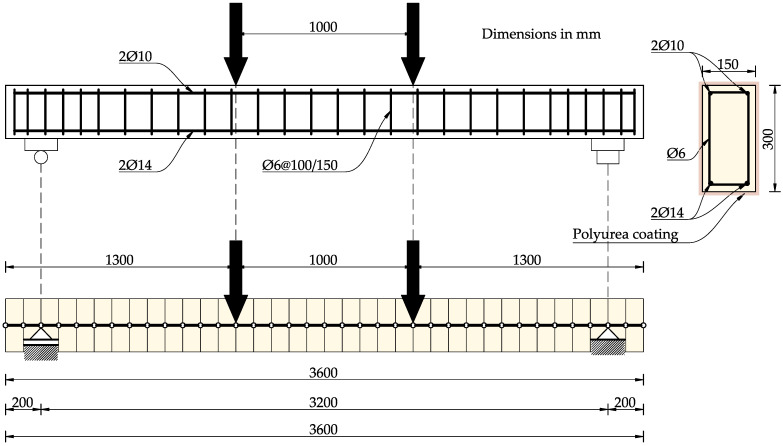
Numerical model of the beam.

Figure 8 shows the static scheme of the beam with the loading forces and boundary conditions.

The length of the element shown in Figure 8 was taken as 3.6 m, and the dimensions of the cross-section were 150 and 300 mm. The beam was divided into 180 finite elements, giving a length of 20 mm for a single finite element. One of the supports was modelled as a pinned support, while the other was modelled as a roller support. Accordingly, the appropriate degrees of freedom were constrained in the corresponding nodes.

The material characteristics for steel and concrete were defined using the UniaxialMaterial ElasticMultiLinear command. This command allows the introduction of any sigma–epsilon characteristics of a nonlinear material, approximating it with a multilinear curve.

**Figure 9:**
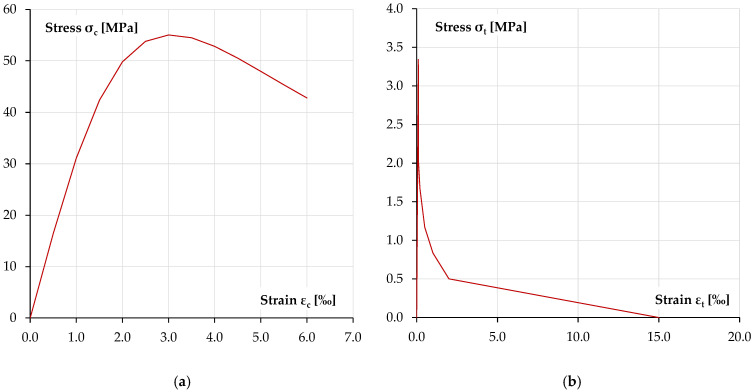
Concrete material models used in numerical analysis: (**a**) material model of concrete in compression [43], (**b**) tension–stiffening model for concrete in tension [44].

**Figure 10:**
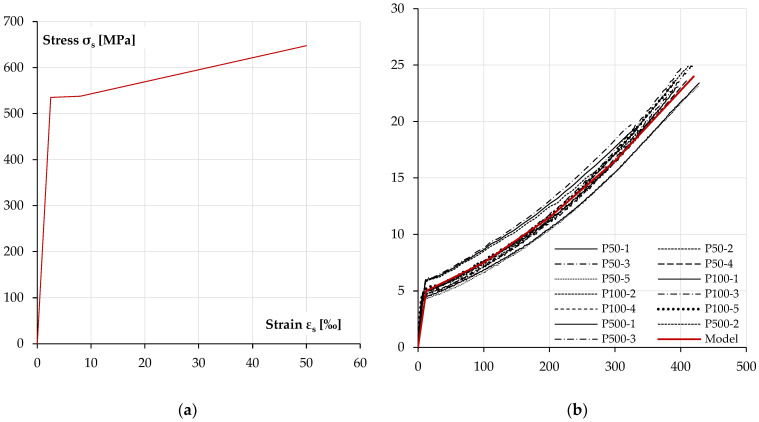
Steel and polyurea material models used in numerical analysis: (**a**) material model of reinforcement steel [46], (**b**) nonlinear relationship of polyurea coating.

Figure 9 shows the material characteristics of concrete in compression and tension adopted in the numerical calculations [43,44]. Figure 10 shows the sigma–epsilon characteristics for the reinforcing steel [46] and the sigma–epsilon characteristic of the polyurea layer, idealised as multilinear on the basis of the provided tests [1].

**Figure 11:**
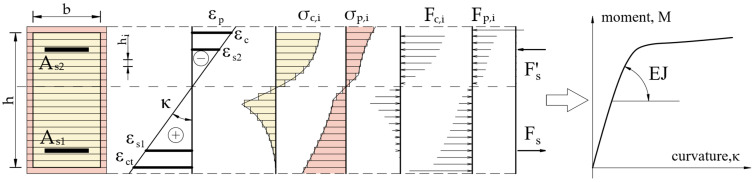
Section layers and the state of strain and stress in the individual layers are used to determine the actual curvature of the section.

Figure 11 shows how the section is divided into layers and the assumed state of deformation and stress in the individual layers of concrete, polyurea and reinforcing steel to determine the current curvature of the section. The cross-section was divided into 200 layers, so the thickness of a single layer was 1.5 mm.

For each integration point, the EJ stiffness is determined directly from the moment–curvature relationship and is identified as the slope of this relationship at the point of the determined curvature.

The nonlinear problem was solved using the Newton–Raphson method. During the calculation, the displacement of the node at the centre of the beam span was controlled. A constant displacement increment of 0.1 mm was adopted. For each increment, a convergence criterion of the solution procedure equal to *ε* = 10^−5^ was applied.

The failure criterion for the beam was assumed to reach 4‰ strain in the extreme compression fibre.

### 4.2. Calculation Results

**Figure 12:**
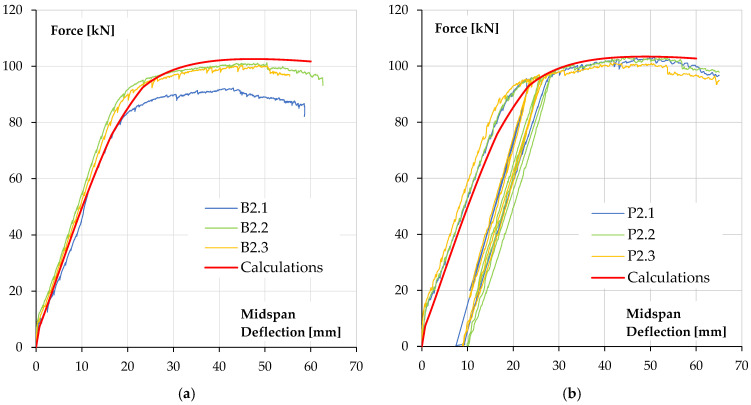
Calculated force–deflection relationships compared to test results: (**a**) reference beams, (**b**) beams with 3 mm polyurea coating.

Figure 12 shows a comparison of the calculated force–deflection relationships with the results obtained in beam tests [1,36,37]. Figure 12a shows the comparison for reference beams without polyurea coating and Figure 12b shows the calculations for beams with a 3 mm polyurea coating.

As can be seen, the agreement of the numerical results with test data is good. Characteristic points on the relationships, such as the force at beam cracking at mid-span and the force at reinforcement steel yield, are in accordance with the experimental data. The ultimate load capacity is also in accordance with the test results.

Table 5 shows a summary of the main test and calculation results of reference beams, and Table 6 shows a summary of the tests and calculations of beams with polyurea coating.

Where terms are defined as follows:
*F_u_*—ultimate load bearing capacity;
*F_cr_*—cracking force;
*F_y_*—force at yielding of the reinforcement steel;
*d*—deflection at midspan.

The numerical calculations confirm the minor influence of the polyurea coating on the behaviour of the presented beams under the load. This is mainly due to the small contribution of the polyurea coating to the flexural stiffness, especially against the background of the relatively high-strength concrete used and the high reinforcement ratio. With the low Young’s modulus of the polyurea and the thin coating layer, the stiffness is basically not increased.

**Figure 13:**
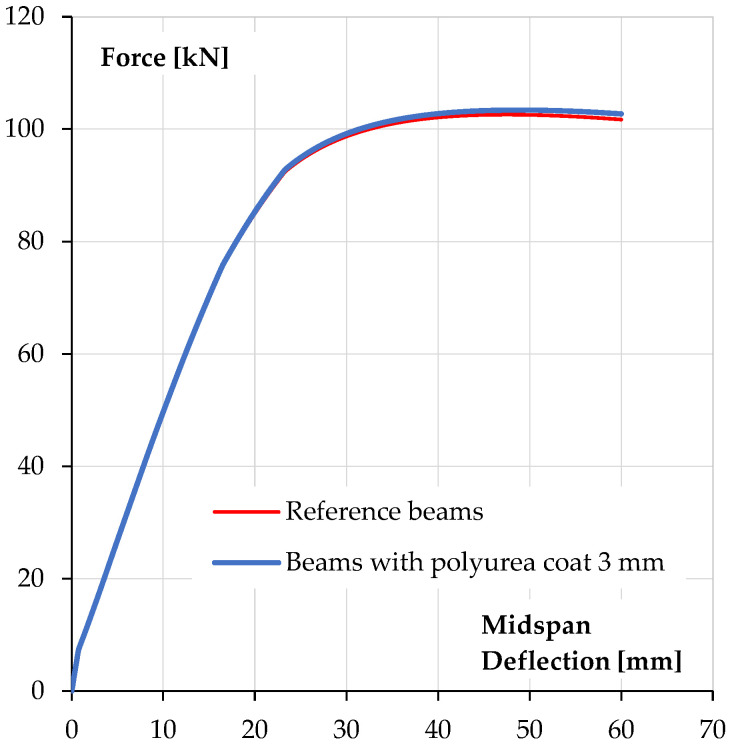
Comparison of calculated force–deflection relationships of reference beams and beams coated with 3 mm polyurea coating.

Figure 13 shows a comparison of calculated relationships for polyurea-coated beams and reference beams. The behaviour under the load, almost over the entire range, i.e., before cracking, after cracking and up to the steel yielding, is the same in both types of beams. The polyurea coating has only a non-significant effect on the behaviour of the beam once the steel reinforcement has yielded; there is a slight increase in post-peak load.

## 5. Parametric Analysis

The effect of the polyurea coating on the load bearing capacity and deformability of bent reinforced concrete elements will also depend on other parameters affecting the flexural stiffness of this type of element. The effect of selected parameters on the behaviour of a reinforced concrete cross-section covered with a polyurea coating is presented below.

### 5.1. Thickness of the Polyurea Coating

**Figure 14:**
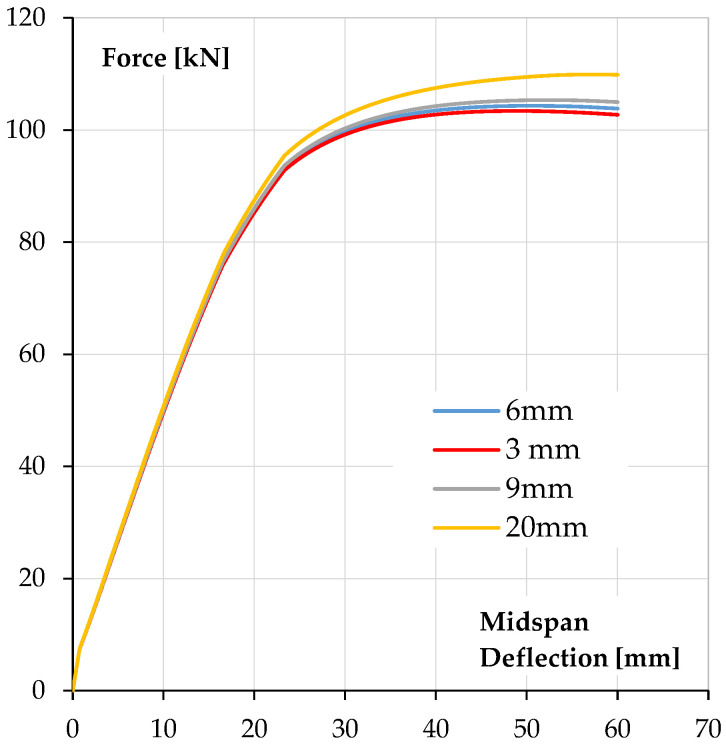
Comparison of calculated force–deflection relationships of beams with different coating thickness.

In the presented research, the thickness of the polyurea was relatively thin. In order to assess the impact of the polyurea coating thickness on the behaviour of the reinforced beam, calculations were carried out on beams with the same geometry and degree of reinforcement as in the studies presented above, but with a thicker coating. Coating thicknesses of 6 mm, 9 mm and 20 mm were assumed. It should be noted that these polyurea coating thicknesses are technically possible but must be applied in several layers. It was assumed that the perfect adhesion of polyurea layers to each other makes it possible to treat thicker layers as a solid. Figure 14 presents the comparison of calculated force–deflection relationships of beams with different coating thickness.

As can be seen, the effect of the polyurea coating on the behaviour under the load changes with increasing coating thickness, but it is limited.

In all cases, the change in coating thickness does not affect the cracking force and flexural stiffness before and after cracking. This effect only becomes apparent in the upper branch of the characteristic relationship, after the reinforcement steel yielding. Here, an increase in load bearing capacity is visible, especially when using the thickest coating, 20 mm (approximately 7%). However, it should still be noted that the increase in load bearing capacity is not significant, especially considering the costs of applying the polyurea coating.

**Figure 15:**
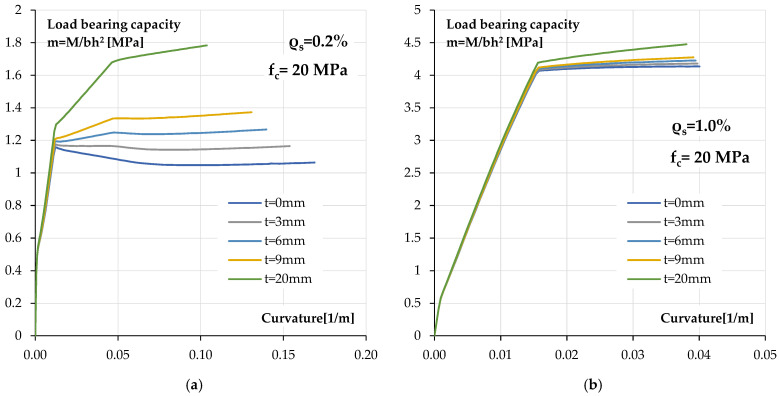
Calculated bending M-κ relationships for reinforced concrete section coated with different polyurea coat thickness: (**a**) low reinforcement ratio *ρ_s_* = 0.2%, (**b**) high reinforcement ratio *ρ_s_* = 1.0%.

Figure 15a shows a summary of the moment–curvature relationships for a reinforced concrete cross-section coated with polyurea with a lower reinforcement ratio than in the tested beams. A reinforcement ratio of *ρ_s_* = 0.2% and a lower concrete class of *f_c_* = 20 MPa were assumed for the calculations. The bending load bearing capacity is presented in dimensionless form m = M/bh^2^.

As can be seen in this case, an increase in load bearing capacity can be expected with an increase in coating thickness. In addition, the use of the coating further stiffens the cross-section after reinforcement steel yielding. This is visible in the form of a change in the slope of the post-critical branch of the moment–curvature relationship. This means higher load bearing capacity of the cross-section, for example, in exceptional situations. For a thicker coating, an additional point on the post-critical branch emerges. It is related to the behaviour of the polyurea coating and its yielding at a strain of around 12.5‰.

For comparison, Figure 15b shows the moment–curvature relationships for a cross-section with a higher reinforcement ratio *ρ_s_* = 1.0%. As can be seen, the effect of covering the cross-section with a polyurea coating decreases and is essentially only significant for the thickest coating with a thickness of 20 mm.

**Figure 16:**
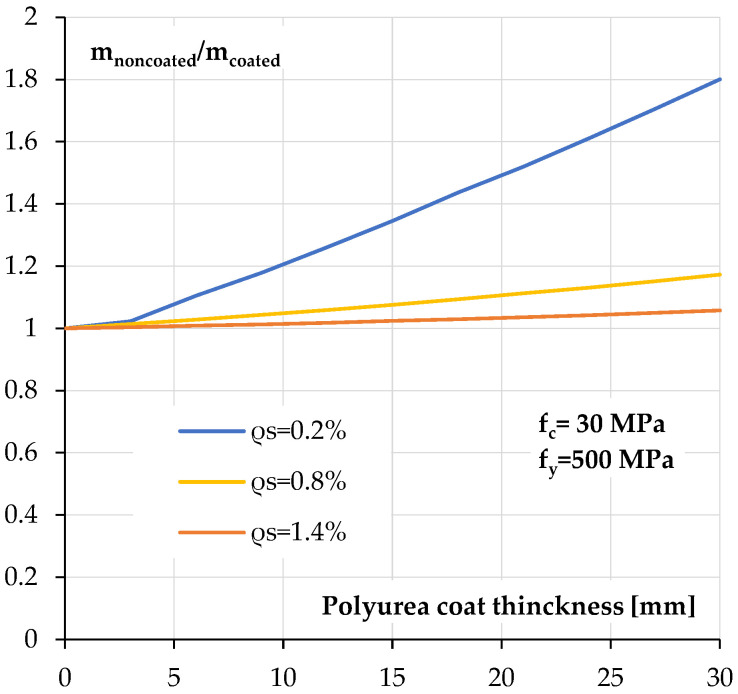
Relationship between calculated load bearing capacity increase and coating thickness of reinforced concrete section.

A more general relationship between the load bearing capacity increase in a reinforced concrete cross-section and the thickness of the polyurea coating is shown in Figure 16. The calculations were performed for two reinforcement ratios of *ρ_s_* = 0.2%, *ρ_s_* = 0.8% and *ρ_s_* = 1.4%.

This relationship confirms earlier conclusions. The effectiveness of the polyurea coating obviously depends on its thickness. The strengthening effect will be greater for sections with a low degree of reinforcement, even with a thinner polyurea coating. For a 6 mm thick coating, an approximately 10% increase in load bearing capacity can be expected, and for a 30 mm thick coating, even 80%.

For sections with a high reinforcement ratio (above 1%), the strengthening effect is not so noticeable; it can be said to be negligible. For thin coatings, the increase in the load bearing capacity of the section is approximately 0.8–1.0%, and for a thickness of 30 mm, it is only 5%. This confirms the findings of beam tests [1].

### 5.2. Reinforcement Ratio

The above considerations confirm that the flexural behaviour of reinforced concrete elements is greatly influenced by the reinforcement ratio. In order to assess the full impact of reinforcement ratio and its interaction with polyurea coating on the behaviour of a polyurea-coated cross-section, a parametric analysis with variable reinforcement ratio was carried out. The calculations were performed for reinforcement ratios ranging from 0.5% to 3.0%. Normal concrete with a strength of *f_c_ =* 30 MPa was assumed, and for comparison, concrete with increased compressive strength of *f_c_ =* 70 MPa was also considered.

**Figure 17:**
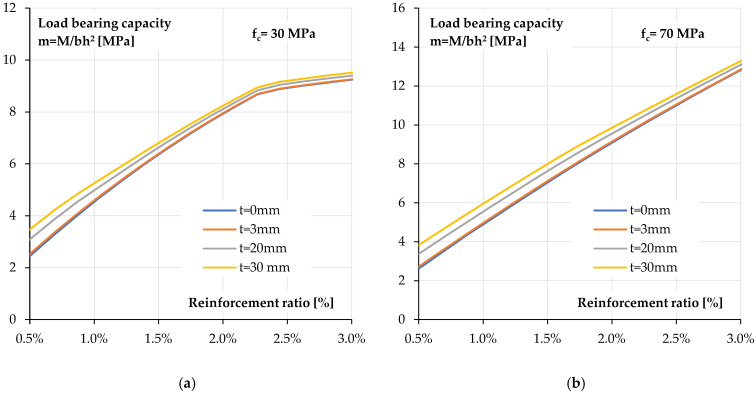
Calculated load bearing capacity—reinforcement ratio relationships for a section coated with different coating thickness: (**a**) concrete compressive strength *f_c_* = 30 MPa, (**b**) concrete compressive strength *f_c_* = 70 MPa.

Figure 17 shows the relationship between flexural load bearing capacity and reinforcement ratio for four coating thicknesses.

For both concrete classes, the reinforcement ratio has a much greater impact than the coating thickness. This is primarily due to the large difference in the modulus of elasticity of those two materials. The effect of the coating application is more noticeable for lower reinforcement ratios and thicker coatings. It disappears as the reinforcement ratio increases. This relationship is similar for both concrete classes.

### 5.3. Reinforcement Steel Characteristic

From a historical perspective, reinforced concrete elements could be reinforced with steel with different, usually lower, yield and ultimate strengths. In Poland, in the post-war years until the early 2000s, steels with a yield strength ranging from *f_y_* = 220 MPa, through to steels with a yield strength of *f_y_* = 355 MPa, and up to 410 MPa, were used. Considering a polyurea coating as a means of protecting and strengthening reinforced concrete elements, it can be assumed that it can be reinforced with steel with a lower yield strength than the steel currently used.

**Figure 18:**
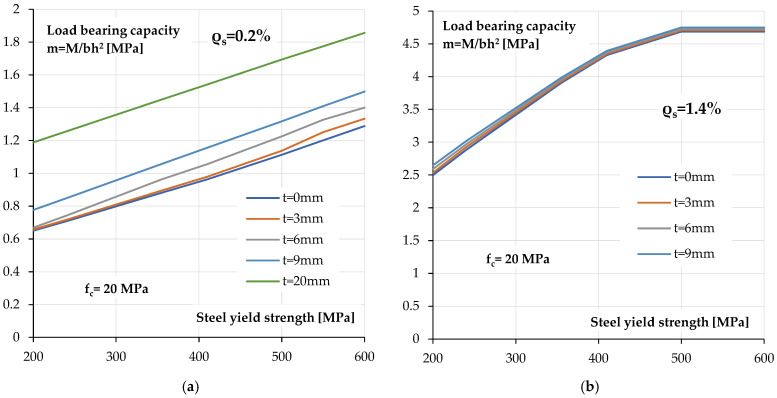
Calculated load bearing capacity—steel yield strength relationships for section coated with different coating thickness: (**a**) low reinforcement ratio *ρ_s_* = 0.2%, (**b**) high reinforcement ratio *ρ_s_* = 1.4%.

Figure 18 shows the relationship between the ultimate load bearing capacity of a reinforced concrete section covered with a polyurea coating and the yield strength of the tensile reinforcement. The calculations were performed for a relatively low concrete class of *f_c_* = 20 MPa, as would be expected in historical elements. In this case, two degrees of reinforcement were assumed *ρ_s_* = 0.2% and *ρ_s_* = 1.4%.

As can be seen for a low reinforcement ratio section (Figure 18a), regardless of the yield strength of the tensile reinforcement used, the increase in load bearing capacity resulting from the use of a polyurea coating is constant. As the thickness of the coating increases, this increase is greater, but the increase in the yield strength has a much greater impact on the load bearing capacity than the coating itself.

For sections with a high reinforcement ratio, the increase in load bearing capacity is also significantly greater with a change in the yield strength of the steel than with a change in the thickness of the coating. The increase in load bearing capacity is minimal due to the increase in coating thickness.

It is worth noting that for a higher yield strength of approximately *f_y_* = 500 MPa, the influence of the compressive strength of concrete on the load bearing capacity of the cross-section becomes apparent. In the relationship in Figure 18b, this is visible as a lack of increase in load bearing capacity with a further change in the yield strength. In this area, the load bearing capacity is compression-controlled.

### 5.4. Concrete Compressive Strength

In order to assess the impact of concrete strength on the ability to reinforce the polyurea coating of a reinforced concrete cross-section, calculations were performed for variable compressive strength. The calculations were performed for reinforced cross-sections with two reinforcement ratios, *ρ_s_* = 0.2% and *ρ_s_* = 1.4%. As in earlier calculations, the polyurea coating thickness was gradually increased.

**Figure 19:**
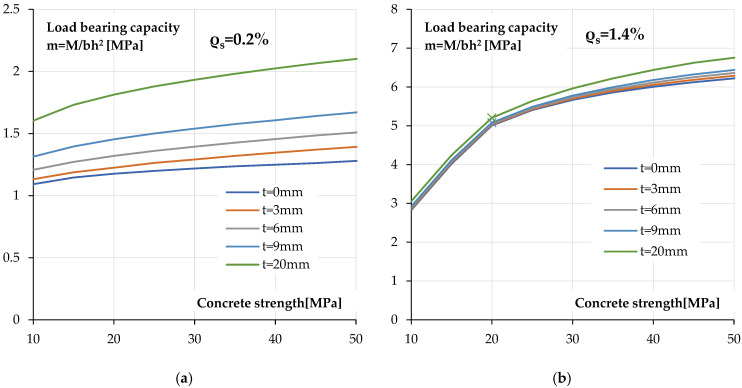
Calculated load bearing capacity—concrete compressive strength relationships for section coated with different coating thickness: (**a**) low reinforcement ratio *ρ_s_* = 0.2%, (**b**) high reinforcement ratio *ρ_s_* = 1.4%.

Figure 19 shows the relationship between the ultimate load bearing capacity and compressive strength of concrete for cross-sections coated with polyurea of varying thicknesses.

Figure 19a shows the relationships for a cross-section with a low reinforcement ratio. As can be seen, the effect of the polyurea coating thickness is visible regardless of the concrete strength used. It can be noted that the increase in load bearing capacity resulting from the use of polyurea is smaller for lower classes. This is due to the earlier utilisation of the compressive strength of low-class concrete.

This is much more evident in Figure 19b, which shows the load bearing capacity and compressive strength relationships for a more heavily reinforced cross-section. In this case, the point of change in the failure mechanism (marked with a cross on relationships) from compression-controlled to tension-controlled is visible as a change in the slope of this relationship.

In this case, as in all previously analysed cases, for a high tensile reinforcement ratio, the increase in load bearing capacity is significantly lower than in the case of cross-sections with a low degree of reinforcement.

## 6. Conclusions

The article examines the influence of polyurea coating on the ultimate load capacity and deformability of reinforced concrete beams. Based on the experimental investigations and the numerical parametric analysis, the following conclusions can be drawn:

- The tests confirm that polyurea coating affects the load bearing capacity and the load-induced behaviour of the beams.
- The effectiveness of the polyurea coating is limited by the material’s relatively low modulus of elasticity.
- An increase in coating thickness enhances the ultimate load bearing capacity but has no significant effect on the pre-yield behaviour of the cross-section.
- The contribution of the coating to the flexural load bearing capacity of the cross-section strongly depends on the longitudinal reinforcement ratio, which proves to be the most influential parameter. The effectiveness of the coating increases as the reinforcement ratio decreases. For heavily reinforced beams, the coating has only a negligible effect.
- The effectiveness of the coating is independent of the type and yield strength of the reinforcing steel. For cross-sections reinforced with low-yield-strength steel, the increase in load bearing capacity due to a thicker coating is comparable to that observed in sections reinforced with normal-yield-strength steel. This trend is observed for both lightly and heavily reinforced sections. In heavily reinforced sections, variations in the yield strength may influence the failure mechanism, potentially shifting the behaviour to compression-controlled failure, in which case the coating’s effectiveness is significantly reduced.
- The influence of concrete compressive strength on coating effectiveness is minor and becomes noticeable primarily for low-strength concretes. In such cases, failure may occur due to crushing of the compressed zone, which limits the coating’s contribution.

The analysis demonstrates that the application of polyurea coatings for structural strengthening is limited, and their use should be preceded by a comprehensive assessment. Both geometric parameters of the coating—primarily its thickness—and, critically, the reinforcement ratio as well as the type of reinforcing steel and concrete must be considered.

Polyurea coatings are a class of elastomeric copolymers which have a unique set of mechanical properties and offer a wide range of applications in construction. The use of polyurea coating in recent decades has been widely practised in modern engineering constructions. It is caused by unusual properties of the coating, such as high elasticity of the material, scratch-resistance, or very high adhesion to many surfaces. The polyurea can be applied to prolong the operation of many existing engineering objects through improving their resistance against external factors (chemical and biological corrosion, or atmospheric factors). The fact that polyurea finds numerous applications across various fields, such as durability and abrasion-resistant systems as well as anticorrosion coatings in tunnels, containers, and vehicle transport spaces, further confirms the above conclusions.

The authors believe that coatings can be used in special cases, e.g., elements requiring both slight reinforcement and strong protection against environmental factors. These may include tanks for aggressive liquids such as biomass, elements exposed to sea salts, acids, alkalis, etc. In these cases, traditional forms of strengthening may not be effective in the planned time frame due to the aggressive environment.

## Figures and Tables

**Table 1:** Basic strength properties of the concrete according to [1,36,37].

-	-	No.	*f_c_*_,*cube*_ */f_ct_*_,*sp*_	Mean
Compressive strength *f_c_*_,*cube*_	MPa	01	71.0	72.3
		02	72.6	
		03	73.5	
Tensile splitting strength *f_ct_*_,*sp*_	MPa	04	3.54	3.60
		05	3.83	
		06	3.50	

**Table 2:** Basic strength properties of top and bottom reinforcing steel according to [1,36,37].

Rebar Diameter	Yielding Strength *f_y_*	Tensile Strength *f_t_*	Young’s Modulus *E_s_*
mm	MPa	MPa	GPa
10	535	648	201
14	509	611	205

**Table 3:** Strength properties of a polyurea coating [1,36,37].

Test Speed	Tensile Strength *f_p_*	Strain *ε_pu_*	Young’s Modulus *E_p_*
(mm/min)	MPa	%	MPa
50	24.1	417	39.9
100	23.3	391	44.7

**Table 4:** Summary of obtained forces and corresponding deflections for reference and polyurea-coated beams according to [1,36,37].

No.	Cross Section	Upper Reinforcement/Lower Reinforcement	Coating Thickness	Cracking Force *F_cr_*	Load Bearing Capacity *F_u_*	Beam Deflection at *F_u_*
-	cm	-	mm	kN	kN	mm
B.2.1	15 × 30	2#10/2#14	-	9.4	92.2	43.0
B.2.2	15 × 30	2#10/2#14	-	11.9	101.2	50.4
B.2.3	15 × 30	2#10/2#14	-	9.5	100.3	48.6
P.2.1	15 × 30	2#10/2#14	2.5–3.0	15.7	103.1	50.7
P.2.2	15 × 30	2#10/2#14	2.5–3.0	14.2	103.2	44.7
P.2.3	15 × 30	2#10/2#14	2.5–3.0	13.54	101.2	42.0

**Table 5:** Comparison of calculations with test results of reference beams.

No.	Beam	*F_u_*, kN	*d*, mm	*F_cr_*, kN	*F_y_*, kN
1	B1.1	92.2	43.0	9.4	80.3
2	B1.2	101.2	50.4	11.9	89.0
3	B1.3	100.3	48.6	9.5	89.6
4	Calculations	102.6	48.0	8.6	90.9

**Table 6:** Comparison of calculations with test results of beams with a 3 mm polyurea coat.

No.	Beam	*F_u_*, kN	*d*, mm	*F_cr_*, kN	*F_y_*, kN
1	P1.1	103.1	50.7	15.7	93.5
2	P1.2	102.1	44.7	14.2	89.1
3	P1.3	101.2	42.0	13.54	87.5
4	Calculations	104.3	50.1	8.6	90.9

## Data Availability

The original contributions presented in this study are included in the article. Further inquiries can be directed to the corresponding author.
